# Hemostatic Achievement After Introduction of Venovenous Extracorporeal Membrane Oxygenation for Severe Multiple Trauma: A Case Study

**DOI:** 10.7759/cureus.25560

**Published:** 2022-06-01

**Authors:** Takahiro Michishita, Kento Nakajima, Tomoki Doi, Kurumi Mori, Ichiro Takeuchi

**Affiliations:** 1 Department of Emergency Medicine, Yokosuka Kyosai Hospital, Yokosuka, JPN; 2 Advanced Critical Care and Emergency Center, Yokohama City University Medical Center, Yokohama, JPN

**Keywords:** venovenous extracorporeal membrane oxygenation, severe trauma, multiple trauma, hemostatic achievement, chest trauma

## Abstract

Venovenous extracorporeal membrane oxygenation (VV-ECMO) is indicated for patients with severe respiratory failure who cannot be managed with a ventilator. We report a case of severe chest trauma with an injury severity score of 66, in which hemostasis was achieved after VV-ECMO. A 29-year-old male patient sustained a fall injury from a 4-m cliff. The fall resulted in significant traumatic cerebral hemorrhage, bilateral pulmonary contusion, hemothorax, pelvic fracture, and limb fracture. During transcatheter arterial embolization, the patient continued to bleed from the left lung and showed progressive hypoxemia. In addition, the patient was unable to maintain tidal volume and experienced hypercapnia, and thus, VV-ECMO was introduced, followed by a thoracotomy to stop the bleeding. On the third day of hospitalization, the patient was weaned off VV-ECMO, and on day 35, he was transferred to a rehabilitation hospital for recovery. VV-ECMO may serve as a “bridge” until hemostatic maneuvers for severe chest trauma are completed and may contribute to help ensure adequate respiration.

## Introduction

Venovenous extracorporeal membrane oxygenation (VV-ECMO) is indicated for patients with severe respiratory failure who cannot be managed with a ventilator. Although VV-ECMO is avoided in patients with trauma because of the bleeding risk, with recent advances in equipment, VV-ECMO has been reported to be useful and safe for patients with trauma [1,2]. An article published in Germany in 2013 introduced ECMO in 52 patients with trauma over a 10-year period, with a survival rate of 79%, which was significantly higher than the 59% injury severity score (ISS)-related mortality rate [1]. A retrospective observational study published in the United States in 2014 reported a significantly improved survival rate in patients with trauma with hypoxemia in the VV-ECMO group compared with that in the mechanical ventilation group [2]. In this report, we describe a case of severe multiple traumas with an ISS of 66 in which VV-ECMO was introduced and consequently hemostasis was achieved.

## Case presentation

History

The patient was a 29-year-old man (height, 177.0 cm; weight, 64.5 kg) with no specific medical history. While touring on a road bike, the patient fell from a 4-m cliff with the bike and was injured. At the time of contact with the paramedics and ambulance team, the patient had ventricular fibrillation and subsequently showed return of spontaneous circulation (ROSC) after one defibrillation, after which he was transported to our hospital. Vital signs after ROSC were as follows: Glasgow Coma Scale (GCS) score, 3 (E1V1M1); blood pressure, 138/89 mmHg; heart rate, 120 beats/min; respiratory rate, 12 breaths/min; SpO_2_, 91% (O_2_ at 10 L/min); pupil diameter, 3 mm/6 mm; and body temperature, 36.0 °C (Table 1).

**Table 1:** Vital summary BP: blood pressure; HR: heart rate; RR: respiratory rate; BT: body temperature; GCS: Glasgow Coma Scale; ROSC: return of spontaneous circulation; FIO_2_: fraction of inspired oxygen

	Pre-hospital	Arrival	After ROSC
Time series (min, after injury)	11	24	36
GCS	3 (E1V1M1)	7 (E1V2M4)	3 (E1VTM1)
Pupil (mm)	3/6	6/6	6/6
BP (mmHg)	138/89	116/71	103/52
HR (beats/min)	120	137	140
RR (breaths/min)	12	32	12
SpO_2_ (%)	91 (O_2 _at 10 L/min)	97 (O_2 _at 10 L/min)	81 (FIO_2_, 1.0)
BT (°C)	36.0	35.6	35.5

After arrival

**Figure 1:**
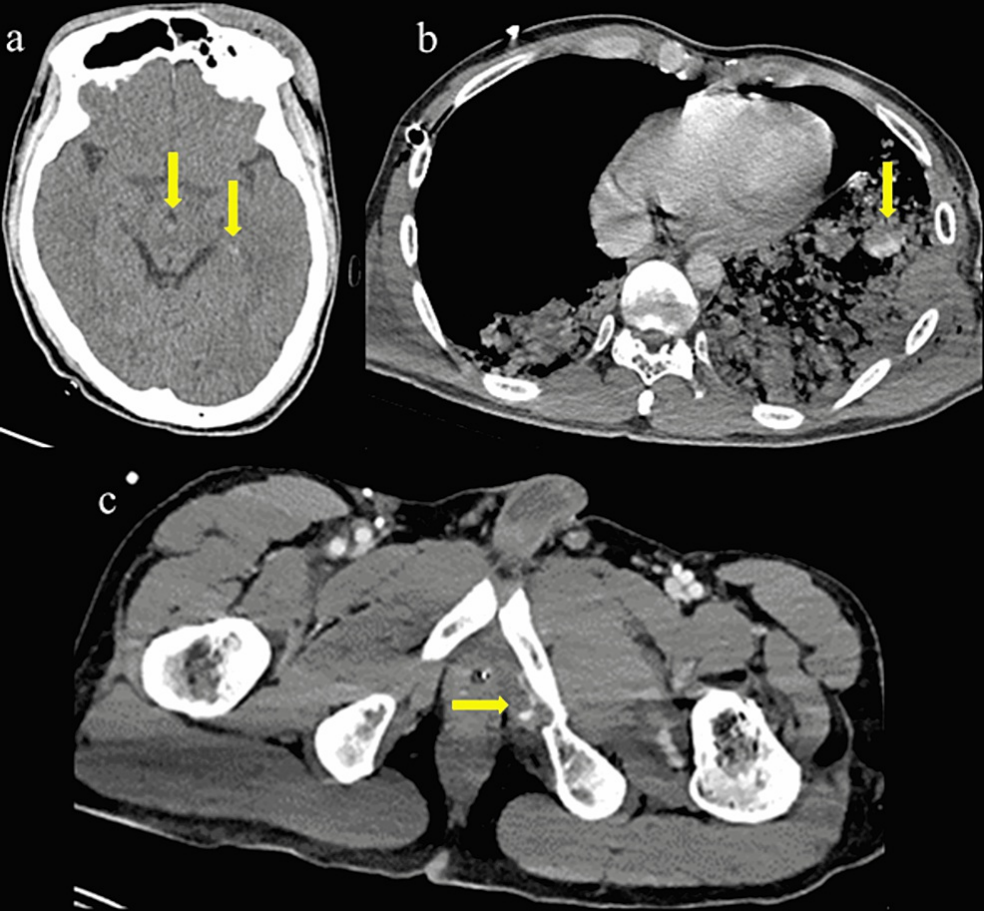
CT findings on admission (a) Head CT showing brainstem hemorrhage and traumatic subarachnoid hemorrhage (AIS code: 140210.5). (b) Chest CT showing active bleeding in the thoracic cavity. (AIS code: 441456.5). (c) Pelvic fracture and active bleeding around the fracture (AIS code 852608.4).

On arrival, the vital signs were as follows: GCS score, 7 (E1V2M4); blood pressure, 116/71 mmHg; heart rate, 137 beats/min; respiratory rate, 32 breaths/min; SpO_2_, 97% (O_2_ at 10 L/min); pupil diameter, 6 mm/6 mm; and body temperature, 35.6 °C (Table 1). Glossoptosis, left-right differences in thoracic movement, and subcutaneous emphysema of the left chest were observed. After arrival, his blood pressure decreased to 55/17 mmHg, and owing to vital signs indicating hypotension and hypoxemia and physical examination findings such as subcutaneous emphysema and left-right differences in thoracic movement, we strongly suspected tension pneumothorax and inserted a left thoracic drain, which revealed degassing and a large amount of bloody drainage. The patient had a GCS score of <8 and was intubated. Although tracheal intubation was performed, the patient experienced cardiopulmonary arrest 10 min after admission, cardiopulmonary resuscitation was initiated, and a left lateral thoracotomy was performed, which caused immediate ROSC after one cardiopulmonary resuscitation cycle. A right thoracic drain was also inserted; however, it did not show degassing or bloody drainage. Blood pressure was stabilized by intravenous administration of extracellular fluids and blood transfusion. Computed tomography (CT) showed brainstem hemorrhage, traumatic subarachnoid hemorrhage, multiple left rib fractures, bilateral pulmonary contusions with left-sided predominance, and bilateral hemothorax. The right thoracic drain did not reach the lung base, and the tip was not positioned at the site of the hemothorax. The pelvic region showed a left sacral fracture and bilateral pubic sciatic fractures. Active bleeding was also observed in the thoracic cavity and from the pelvic fractures (Figures 1a-1c).

**Figure 2:**
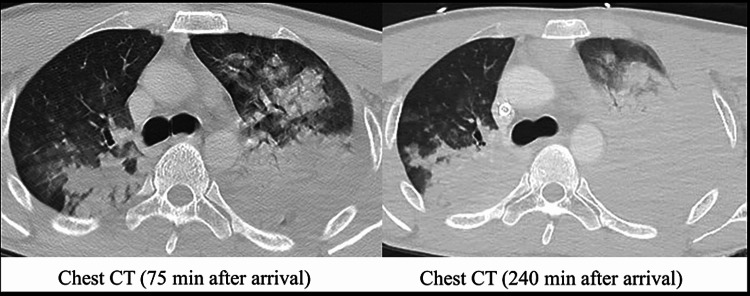
Chest CT showing lung air content due to intrathoracic hemorrhage at 240 min after arrival (right) was markedly lower than that at 75 min after arrival (left).

**Figure 3:**
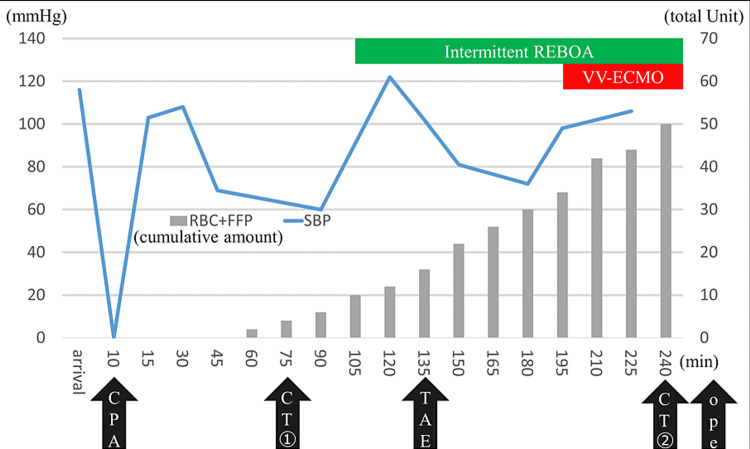
Course summary after arrival A total of 50 units of blood was transfused at 240 min from the time of arrival. CPA: cardiopulmonary arrest; RBC: red blood cell (1 U, 80 mL); FFP: fresh frozen plasma (1 U, 70 mL); SBP: systolic blood pressure; TAE: transcatheter arterial embolization; Ope: operation; REBOA: resuscitative endovascular balloon occlusion of the aorta; VV-ECMO: venovenous extracorporeal membrane oxygenation

The ISS was 66 (based on the AIS90 Update 98), the revised trauma score was 2.3376, and the probability of survival was 0.01677, indicating severe multiple traumas. After undergoing CT, the patient went into shock again (systolic blood pressure, 60 mmHg; heart rate, 150 beats/min; shock index, 2.5). Considering bleeding from a pelvic fracture as the main site of hemorrhage, resuscitative endovascular balloon occlusion of the aorta (REBOA, RESCUE BALLOON®-ER) catheter was inserted in aortic zone 1, and his blood pressure increased to 122/44 mmHg. The patient underwent transcatheter arterial embolization (TAE) for active bleeding at the pelvic fracture site and was scheduled for thoracotomy to stop the bleeding. During TAE, the left thoracic cavity continued to bleed, and the blood drainage of the left thoracic drain reached 1,000 mL. The hypoxemia worsened to a partial pressure of arterial oxygen (PaO_2_)/fraction of inspired oxygen (FIO_2_) ratio of 64.0, the tidal volume was not maintained, PaCO_2_ increased to 52.6 torr (pH, 7.287), and the progression of acidemia could not be controlled. The respiratory condition was unsustainable until thoracotomy, VV-ECMO (right femoral vein decannulation, 23 Fr; right internal jugular vein pumping, 21 Fr) was introduced in the fluoroscopy room without heparin, and ECMO flow was maintained at a minimum of approximately 3.7 L/min at 1,500 rpm. After initiation of VV-ECMO, the second CT was performed (Figure 2). After completion of TAE, the patient underwent thoracotomy in the operating room and was admitted to the intensive care unit (ICU) after hemostasis of the intercostal arteriovenous system around the rib fracture. By the time he was admitted to the ICU, 44 U of red blood cells, 60 U of fresh frozen plasma, and 40 U of platelet concentrate (PC) had been transfused (Figure 3).

After hospitalization

Bronchoscopy was performed on day 3, and blood clots and bloody sputum were aspirated. After suctioning, the lung compliance increased from 25 to 50 mL/cmH_2_O, and the tidal volume increased from 250 to 500 mL; the flow of VV-ECMO was turned off; oxygenation and ventilation capacity were maintained by self-lung (PaO_2_/FIO_2_ ratio, 250 mmHg; PaCO_2_, 41 torr); and VV-ECMO was removed. During his hospitalization, there was no exacerbation of the cerebral hemorrhage; tracheostomy was performed on day 7, and the patient was transferred from the ICU to a general bed on day 17. On day 35, the patient was transferred to a rehabilitation hospital for recovery. Eight months after the injury, the patient had higher brain dysfunction due to diffuse axonal injury but was able to take food orally, communicate with others, and undergo gait training.

## Discussion

In this case, the patient presented with hypoxemia and ventilatory failure due to chest trauma, and VV-ECMO was introduced to improve hypoxemia and hypercapnia. It is believed that hemostatic maneuvers are important in trauma, and VV-ECMO may be useful to ensure respiration.

VV-ECMO is a useful option for severe respiratory failure that is difficult to manage only on a ventilator. ECMO is usually not indicated in patients with trauma because of the bleeding risk. In previous reports, VV-ECMO has been used in some cases of life-threatening hypoxemia due to trauma, including head injury [3-5]. In this case, bleeding was not controlled even before VV-ECMO was introduced, and ECMO was established without anticoagulation. When ECMO is introduced for hypovolemic shock, intravascular volume must be maintained to keep adequate flow, and large-volume transfusion should be performed if necessary. Fifty units of blood were transfused within 4 h of admission, and ECMO flow was maintained at a minimum of approximately 3.7 L/min at 1,500 rpm (Figure 3).

In this case, the patient showed bilateral pulmonary contusion and massive hemothorax, hypoxemia, and ventilatory failure due to atelectasis associated with inhalation and stagnation, which necessitated the introduction of VV-ECMO. The second CT image in the primary care unit showed a significant reduction in the lung air content (Figure 2).

In previous studies, VV-ECMO was indicated after trauma with a PaO_2_/FIO_2_ ratio of <80 mmHg, a maximum positive end-expiratory pressure (PEEP) of 18 cmH_2_O, and persistent respiratory acidosis (pH, <7.25) [1,2]. The patient had hypoxemia (PaO_2_/FIO_2_ ratio, 62.4 mmHg) and the tidal volume was not maintained, PaCO_2_ increased to 52.6 torr (pH, 7.287), and the progression of acidemia could not be controlled just before the introduction of VV-ECMO. VV-ECMO was introduced for severe hypoxemia and respiratory acidosis.

During TAE, the patient was in a state of prolonged hypovolemic shock due to continued bleeding and circulatory instability; therefore, PEEP could not be increased, and there was no time to perform bronchoscopy. At the time of thoracotomy, the injury to the lung parenchyma was mild, and no lobectomy was indicated. After proper hemostatic maneuvers, the circulation was stable, and the bronchoscopy could be performed on day 3 and weaned off VV-ECMO. Thus, VV-ECMO was introduced to improve hypoxemia and hypercapnia as a strategy against trauma.

This patient had an ISS of 66 and severe multiple traumas. This ISS is the highest among all reported survival cases of chest trauma treated with VV-ECMO [1-3,6-8]. A major factor in the patient’s survival was the introduction of VV-ECMO as a “bridge” for proper hemostatic maneuvers and the completion of proper hemostasis. As a result, the patient was transferred to a rehabilitation hospital with no exacerbation of cerebral hemorrhage and an improving neurological prognosis.

## Conclusions

In this case, the patient presented with hypoxemia and ventilatory failure due to chest trauma, and VV-ECMO was successfully applied to improve hypoxemia and hypercapnia. ECMO is not a common option in patients with trauma because of the bleeding risk. However, it is believed that hemostatic maneuvers are important in trauma. This case study shows that VV-ECMO may serve as a “bridge” until hemostatic maneuvers for severe chest trauma are completed and may contribute to help ensure adequate respiration.
